# Induction of γδT cells from HSC‐enriched BMCs co‐cultured with iPSC‐derived thymic epithelial cells

**DOI:** 10.1111/jcmm.16993

**Published:** 2021-10-23

**Authors:** Naoki Hosaka, Seiji Kanda, Takaki Shimono, Toshimasa Nishiyama

**Affiliations:** ^1^ Department of Pathology Fuchu Hospital Izumi Osaka Japan; ^2^ Department of Hygiene and Public Health Kansai Medical University Hitakata Osaka Japan; ^3^ Regenerative Research Center for Intractable Diseases Kansai Medical University Hitakata Osaka Japan

**Keywords:** bone marrow cells, induced pluripotent stem cells, thymic epithelial cells, γδT cells

## Abstract

T cells bearing γδ antigen receptors have been investigated as potential treatments for several diseases, including malignant tumours. However, the clinical application of γδT cells has been hampered by their relatively low abundance *in vivo* and the technical difficulty of inducing their differentiation from hematopoietic stem cells (HSCs) *in vitro*. Here, we describe a novel method for generating mouse γδT cells by co‐culturing HSC‐enriched bone marrow cells (HSC‐eBMCs) with induced thymic epithelial cells (iTECs) derived from induced pluripotent stem cells (iPSCs). We used BMCs from CD45.1 congenic C57BL/6 mice to distinguish them from iPSCs, which expressed CD45.2. We showed that HSC‐eBMCs and iTECs cultured with IL‐2 + IL‐7 for up to 21 days induced CD45.1^+^ γδT cells that expressed a broad repertoire of *V*γ and *V*δ T‐cell receptors. Notably, the induced lymphocytes contained few or no αβT cells, NK1.1^+^ natural killer cells, or B220^+^ B cells. Adoptive transfer of the induced γδT cells to leukemia‐bearing mice significantly reduced tumour growth and prolonged mouse survival with no obvious side effects, such as tumorigenesis and autoimmune diseases. This new method suggests that it could also be used to produce human γδT cells for clinical applications.

## 1 INTRODUCTION

Adoptive transfer of *in vitro*‐expanded patient‐derived tumour antigen‐specific αβT cells was one of the earliest immune therapies for cancer. Since then, approaches using adoptive transfer of αβT cells bearing engineered T‐cell receptors (TCRs) have achieved some success but have other limitations.^1^, ^2^ More recently, interest has renewed in the use of γδT cells for various clinical applications, including cancer.^3^, ^4^ γδT cells display potent cytotoxicity but differ from classical αβT cells in several aspects. They develop earlier than αβT cells, account for only 2%–3% of T cells in lymphoid organs,^5^, ^6^ and are predominant in the intestine, skin and mucosa.^7^ Moreover, γδTCRs predominantly recognize mycobacterial isopentenyl pyrophosphate and related prenyl pyrophosphate derivatives in a major histocompatibility complex‐independent manner^8^ and have functional properties that overlap those of innate immune cells and adaptive αβT cells.^9^, ^10^ Accordingly, γδT cells play crucial roles not only in the immune response to infection but also in anti‐tumour immunity,^11^ although the precise mechanisms underlying their anti‐tumour activity remain unclear.

Because of the relative scarcity of γδT cells *in vivo*, boosting their production has been difficult in immunosuppressed patients who may benefit from increased numbers of γδT cells, such as in patients who have undergone chemoradiation therapy, have received immunosuppressive drug treatment or have acquired/congenital immune deficiency syndromes. Whilst several methods have been described for the large‐scale *ex vivo* and *in vitro* expansion of αβT cells, similar attempts to expand γδT cells have met with little success. However, recent advances in regenerative medicine have increased our ability to manipulate hematopoietic stem cells (HSCs) and induce pluripotent stem cells (iPSCs) to generate T cells *in vitro*, although current methods are usually complex and incompletely reliable.

T cells develop naturally in the thymus, and their differentiation from HSCs is critically dependent on thymic epithelial cells (TECs).^12^, ^13^ Indeed, thymus transplantation has shown benefit in the treatment of several intractable diseases, including autoimmune diseases and malignant tumours.^14^, ^15^, ^16^ However, obtaining sufficient thymus tissue for clinical application is difficult, and the function is the highest in neonates and decreases with age.^17^, ^18^ Therefore, differentiation of TECs from iPSCs *in vitro* has been proposed as a possible approach to overcome this supply issue. Embryonic stem cells (ESCs) and tissue stem cells (TSCs)^19^ have been successfully employed to generate mouse and human TECs. However, ethical problems are associated with the use of human ESCs and obtaining sufficient TSCs from clinical specimens is difficult. Therefore, we considered the potential use of iPSCs to generate TECs *in vitro*.^20^

In the current report, we describe a new method to generate γδT cells *in vitro* through co‐culture of HSCs enriched from bone marrow cells (HSC‐eBMCs) with induced TECs (iTECs) differentiated from iPSCs. We demonstrate that the generated γδT cells express a broad TCRγδ repertoire and have anti‐tumour activity in a mouse leukemia model but do not lead to autoimmunity or *de novo* tumorigenesis *in vivo*. Our findings suggest that this method, which employs readily available BMCs and iPSCs, could also be applied to generate therapeutic anti‐tumour γδT cells in humans.

## 2 MATERIALS AND METHODS

### 2.1 Mice and cell lines

C57BL/6 mice (H‐2^b^, CD45.2^+^) were purchased from Shimizu Experimental Animal Laboratory (Shizuoka, Japan), and congenic CD45.1^+^ C57BL/6 mice were purchased from RIKEN BioResource Center (Ibaraki, Japan). All animals were maintained under specific pathogen‐free conditions. Mouse iPSCs were provided by RIKEN BioResource Center.^21^ Cells were cultured in advanced Dulbecco's modified Eagle medium (Thermo Fisher Scientific, Waltham, MA, USA) supplemented with 50% supernatant from mouse foetal fibroblasts in Dulbecco's modified Eagle medium (Sigma‐Aldrich, St. Louis, MO, USA) with 10% fetal bovine serum. Cells were cultured in the presence of 10^3^ U/ml leukemia inhibitory factor (ORF Genetics Ltd., Kópavogur, Iceland) and 0.1 mM 2‐mercaptoethanol (2ME) (Sigma‐Aldrich) in 0.1% gelatin from bovine skin (Sigma‐Aldrich)‐coated 10‐cm dishes. They were passaged every 2 or 3 days by treatment with 0.25%Trypsin‐EDTA (Thermo Fisher Scientific) for 1 min at 37°C. Aliquots of cells in the proliferative phase were harvested and maintained in liquid nitrogen until use in experiments. The Animal Experimentation Committee of Kansai Medical University reviewed and approved the animal studies. EL‐4 (H‐2^b^, CD45.2^+^), a leukemic T‐cell line derived from C57BL/6 mice,^22^, ^23^ was used for the *in vivo* study.

### 2.2 Induction of iTECs from iPSCs

iTECs were differentiated from iPSCs using a modification of the method reported previously.^20^ Briefly, aliquots of 2 × 10^5^ iPSCs were cultured in 10 ml SF‐3 (Sanko Junyaku, Tokyo, Japan) supplemented with 0.1 mM 2ME, 5 mM LiCl (Sigma‐Aldrich), and 10 ng/ml activin A (R&D Systems, Minneapolis, MN, USA) in 10‐cm type IV collagen‐coated dishes (Nitta Gelatin, Osaka, Japan). On day 4, the medium was replaced with SF‐3 containing 0.1 mM 2ME, 5 mM LiCl, 4 ng/ml fibroblast growth factor‐8 (FGF8; R&D Systems) and 5 ng/ml activin A. On day 7, the medium was replaced with SF‐3 containing 0.1 mM 2ME, 5 mM LiCl, 20 ng/ml FGF7 (R&D Systems), 10 ng/ml FGF10 (R&D Systems) and bone morphogenetic protein‐4 (BMP4; R&D Systems) and the incubation was continued for 3 weeks. The adherent cells (iTECs) were harvested by treatment with 0.25% trypsin‐EDTA for 2 min at 37°C for analysis.

### 2.3 RT‐PCR analysis

Total RNA was extracted from cells using TRIzol (Thermo Fisher Scientific), and aliquots of 1 µg RNA were reverse‐transcribed using ReverTra Ace (Toyobo, Osaka, Japan). PCR was performed using Tks Gflex DNA polymerase (Takara Bio, Shiga, Japan) with reaction conditions of 30 cycles of denaturation at 94°C for 1 min, annealing at 55°C for 15 s and elongation at 68°C for 30 s. PCR primers are listed in Table S1. *TCR Vγ* or *Vδ* sense primers were used together with *common γ* or *common δ* antisense primers, respectively, as previously described.^24^, ^25^ PCR products were analysed by electrophoresis on 1.5% agarose gels and visualized by ethidium bromide staining.

### 2.4 *CD45*.*1* and *CD45*.*2* allele typing by PCR

PCR primers used in this analysis are listed in Table S2. Sense primers for *CD45*.*1 (Ly5*.*1)* or *CD45*.*2 (Ly5*.*2)* were used together with a *common CD45 (Ly5)* antisense primer. Briefly, genomic DNA was extracted from iPSCs or splenocytes from CD45.1 and CD45.2 congenic C57BL6 mice using a DNeasy Blood and Tissue Kit (Qiagen, Hilden, Germany). PCR was performed using ExTaq DNA polymerase (Takara Bio) with reaction conditions of 30 cycles of denaturation at 98°C for 10 s, annealing at 55°C for 30 s and elongation at 72°C for 1 min. PCR products were analysed by electrophoresis on 1.5% agarose gels and visualized by ethidium bromide staining.

### 2.5 Enrichment of HSCs from BMCs

BM was flushed from the shafts of femurs and tibias from 6‐ to 8‐week‐old CD45.1^+^ C57BL/6 congenic mice, and single‐cell suspensions were prepared by passaging through a 70‐μm nylon cell strainer (Corning, Corning, NY, USA). Low‐density BM mononuclear cells (MNCs) were isolated by Ficoll‐Paque PLUS density gradient centrifugation (<1.077 g/ml; GE Healthcare, Uppsala, Sweden) to exclude granulocytes and red blood cells. Lineage‐positive cells expressing CD3, CD4, CD8 (T cells), CD11b (myeloid cells), B220 (B cells), Gr‐1 (macrophage/monocytes), CD71, TER119 (erythroid cells) or NK1.1 (natural killer cells) were removed from the MNCs by incubation with a rat anti‐mouse monoclonal antibody (mAb) cocktail as above (BD Biosciences, Franklin Lakes, NJ, USA), followed by incubation with sheep anti‐rat IgG‐conjugated magnetic beads (Dynal Inc., Oslo, Norway) with gentle agitation according to the manufacturer's protocol. Bead‐bound cells were removed using a magnetic particle concentrator (Dynal Inc.). The remaining lineage‐negative MNCs were considered as HSC‐eBMCs.

### 2.6 Co‐culture of HSC‐eBMCs and iTECs to produce induced lymphocytes (iLs)

HSC‐eBMCs (1 × 10^5^) were added to culture dishes containing iTECs produced as described above for 14 days. The medium was changed to fresh SF‐3 supplemented with 0.1 mM 2ME with 20 ng/ml IL‐2 (PeproTech Inc., Rocky Hill, NJ, USA) and 10 ng/ml IL‐7 (PeproTech Inc.). For experiments lacking iTECs, HSC‐eBMCs were cultured in 10‐cm type IV collagen‐coated dishes. The cultured cells containing iLs were collected after 7, 14 and 21 days in culture, passed through a 70‐µm nylon cell strainer and analysed.

### 2.7 Flow cytometry

Cells were analysed by four‐colour immunofluorescence staining using a FACSCalibur cytometer (BD Biosciences). Anti‐mouse CD45.1, CD45.2, TCRβ, TCRγδ, CD3, CD4, CD8, CD34, CD117 (c‐kit), B220 and NK1.1 mAbs coupled to fluorescein isothiocyanate (FITC), phycoerythrin (PE), PerCP/Cy5.5 or allophycocyanin (APC) (BioLegend, San Diego, CA, USA) were used to identify lymphocyte subsets. Data were analysed with FlowJo software (BD Biosciences).

### 2.8 Autologous bone marrow transplantation (BMT) and transfer of iLs

Recipient CD45.2^+^ C57BL/6 mice were lethally irradiated (8 Gy) using a ^137^Cs irradiator (Gammacell 40 Exactor irradiator; MDS Nordion International, Ottawa, ON, Canada). Five hours later, the irradiated mice were injected intravenously with a single‐cell suspension of BMCs (1 × 10^7^) isolated from donor CD45.2^+^ C57BL/6 mice. The mice were injected intravenously with 3 × 10^6^ iLs harvested from co‐cultures of HSC‐eBMCs +iTECs + IL‐2 + IL‐7 on day 7. Spleen, peripheral blood, small intestine lymph nodes, thymus and bone marrow were harvested from the mice 14 days later, and CD45.1^+^ iLs were identified and analysed by flow cytometry. For histological analysis, liver, lung, kidney and small intestine were harvested on day 31 after iL injection.

### 2.9 Mouse leukemia model and adoptive transfer of iLs

For analysis of the anti‐tumour effects of iLs, iLs were harvested from co‐cultures of HSC‐eBMCs +iTECs + IL‐2 + IL‐7 on day 7. iLs was depleted of γδT cells by incubation with avidin‐conjugated anti‐TCRγδ mAb GL3 (BioLegend) and biotin‐conjugated immunobeads (Dynal Inc.). The presence of residual γδT cells in the depleted cell preparation was evaluated using FITC‐conjugated anti‐TCRγδ mAb UC7 (BioLegend). C57BL/6 mice were injected with 1 × 10^6^ EL‐4 (CD45.2^+^ H‐2^b^) leukemic cells and then with iLs or γδT cell‐depleted iLs (3 × 10^6^).^22^ Liver, lung, kidney and spleen were harvested on day 50 post‐injection and analysed for the presence of leukemic cells and evidence of autoimmunity and tumorigenesis. The survival of additional groups of mice was monitored for up to 10 weeks.

### 2.10 Histology

Mouse tissues were fixed in 10% formaldehyde and embedded in paraffin. Four‐micrometre‐thick sections were prepared and stained with hematoxylin and eosin (H&E).

### 2.11 Statistical analyses

Non‐parametric analyses (Mann‐Whitney U test and log‐rank test) were performed using StatView (Abacus Concepts, Berkley, CA, USA). *p* < 0.05 was considered statistically significant.

## 3 RESULTS

### 3.1 Induction of TECs from iPSCs

**FIGURE 1:**
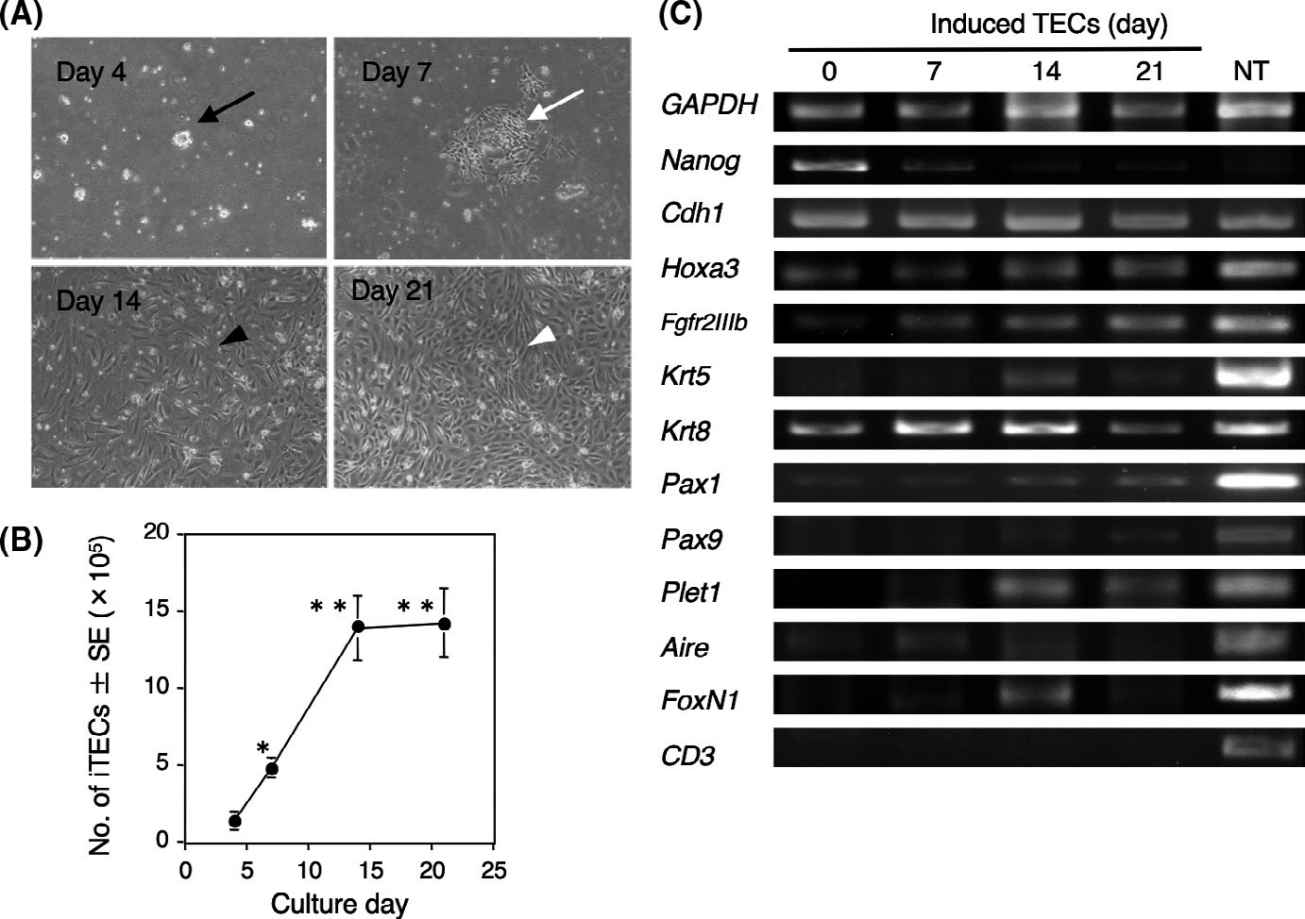
Induction of TECs from iPSCs. (A) Morphology of cells after incubation of iPSCs with activin A, FGFs and BMP4for up to 21 days. Black arrow indicates a small cell mass, white arrow indicates the emergence of spindle epithelial‐like cells, and black and white arrowheads indicate increasing of the cells and the dense structure. (B) Number of iTECs during 3 weeks of culture shown in (A). Data are the mean ±SEM of *n* = 4–5 replicates. *p < 0.05 versus day 4, **p < 0.05 versus days 4 and 7 by the Mann‐Whitney U test. (C) RT‐PCR analysis of gene expression profiles of iPSCs (day 0) and iTECs after 7, 14 and 21 days in culture. Mouse newborn thymus (NT) were analysed as a control

To determine whether γδT cells could be generated *in vitro* from HSC‐eBMCs and iTECs, we first investigated the culture conditions required for induction of iTECs. Using a method modified from an earlier report,^20^ iPSCs were cultured in medium containing activin A and LiCl to induce mesendodermal differentiation. After 4 days, the medium was exchanged to contain FGF8 and activin A to induce mesendodermal to endodermal transition and early differentiation of epithelial cells. On day 7, the medium was exchanged to contain FGF7, FGF10 and BMP4 to induce the proliferation of epithelial cells. Light microscopy revealed small masses of cells by day 4 (Figure 1A), followed by the emergence of small numbers of spindle epithelial‐like cells by day 7, increasing the close proliferation of the cells by day 14 and the maintenance of the dense structure between days 14 and 21. The number of the cells increased between day 7 and day 14 and plateaued thereafter (Figure 1B).

We examined the expression of several critical transcription factors associated with haematopoiesis by RT‐PCR analysis of iPSCs and iPSC‐derived cells on days 7, 14 and 21 (Figure 1C). Expression of *Nanog*, which is a pluripotency marker,^26^ was apparent on day 0, as expected, and then decreased on day 7 and was virtually undetectable thereafter. Conversely, expression of the epithelial marker E‐cadherin *(Cdh1)* and *Hoxa3*, a marker of initial thymus formation,^27^ was detectable at all time points. Expression of the FGF receptor *Fgfr2IIIb*, which binds to FGFs and signals for induction of TECs,^28^ gradually increased between day 0 and day 21. The medullary TEC marker keratin 5 *(Krt5)* was detected only on day 14, whilst the cortical TEC marker *Krt8* was detected at all time points. *Pax1* and *Pax9*, which lie downstream of *Hox3* during thymus formation,^27^, ^29^, ^30^ and *Plet1*, a marker for early TEC progenitors,^31^ were detected after day 14, albeit at low levels. Autoimmune regular *(Aire)*, which is expressed in medullary TECs and regulates the expression of tissue‐specific antigens,^32^ was detected from day 0 to day 21. Finally, *Foxn1*, a marker of mature TECs,^33^ was expressed on day 14 and, to a lower extent, on day 21. The T‐cell marker *CD3* was barely detectable on all days analysed. These findings, which were similar to those obtained in a previous study of the generation of iTECs from iPSCs *in vitro*,^20^ suggest that TECs can be induced from iPSCs, and that iTECs are virtually terminally differentiated after day 14.

### 3.2 iPSCs express the *CD45*.*2* allele exclusively

Next, we examined the ability of iTECs to induce T‐cell development from BMCs. Because iPSCs can also differentiate into T cells, we exploited the fact that the iPSCs expressed the *CD45*.*2* allele whilst the BMCs were prepared from CD45.1^+^ congenic mice (Figure S1). Thus, BMC‐derived cells and iPSC‐derived cells could be distinguished by expression of CD45.1 and CD45.2, respectively.

### 3.3 Generation of HSC‐eBMCs

To generate HSCs enriched in BMCs, single‐cell suspensions of BM MNCs from C57BL/6 congenic CD45.1^+^ mice were depleted of cells of the T, B, granulocyte, erythrocyte, macrophage/monocyte and NK cell lineages by negative selection with mAbs against CD3, CD4, CD8, B220, Gr‐1, CD11b, TER119, CD71 and NK1.1. The percentage of lineage‐negative HSCs (CD34^+^ and CD117 [c‐kit]^+^) after depletion was approximately 30% compared with 1% of the original BMCs (Figure S2).^34^, ^35^ Notably, the HSC‐eBMC preparation contained <0.5% TCRγδ^+^ and TCRαβ^+^ cells with low CD45.1 expression, which compared with approximately 2%–5% TCRγδ^+^ and TCRαβ^+^ cells with high CD45.1 expression in the input BMC preparation. These results indicate that the HSC‐eBMCs were approximately 30‐fold enriched in HSCs and contained few mature T cells.

### 3.4 Generation of iLs by co‐culture of HSC‐eBMCs and iTECs

**FIGURE 2:**
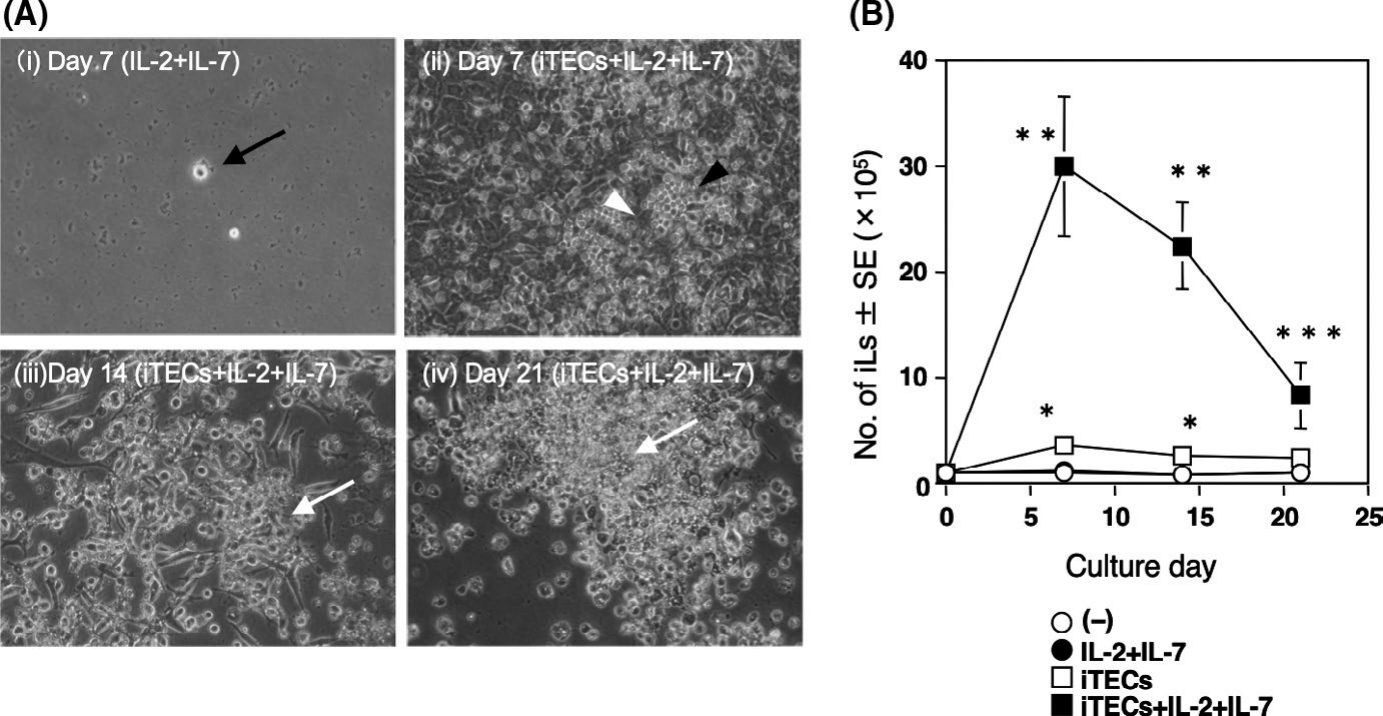
Generation of induced lymphocytes (iLs) by co‐culture of HSC‐eBMCs and iTECs. (A) Morphology of emerging iLs after incubation of HSC‐eBMCs with iTECs, IL‐2 and IL‐7 for up to 21 days. Black arrow indicates a single lymphocyte on day 7 (IL‐2 + IL‐7). Black arrowhead indicates an iL in contact with iTECs (white arrowhead) on day 7, and white arrows indicate increasing death of iLs on days 14 and 21(iTECs +IL‐2 + IL‐7). (B) Number of iLs in cultures of HSC‐eBMCs alone (white circles), HSC‐eBMCs with IL‐2 + IL‐7 (black circles), HSC‐eBMCs with iTECs (white squares) and HSC‐eBMCs with iTECs +IL‐2 + IL‐7 (black squares). Data are presented as the mean ±SEM of *n*=4–5 replicates. *p < 0.05 versus ○, ●; **p < 0.05 versus ○, ●, □; ***p < 0.05 versus ○, ● by the Mann‐Whitney U test. ^#^ Symbols (●) are almost directly behind the symbols (○), because the results of the former were almost same as those of the latter

Next, we examined induction of lymphocyte differentiation by co‐culture of 1 × 10^5^ HSC‐eBMCs with iTECs in the same dish in the presence of IL‐7, which induces differentiation of HSCs into lymphocyte precursors, and IL‐2, which induces the development of the precursors into cells of the T lymphocyte lineage. The co‐culture conditions were determined according to the maturation of the iTECs at day 14 to preserve their structure, because the microenvironment of TECs is crucial for the generation of T cells in the thymus.^36^ The number of iTECs was approximately 1.4 × 10^6^ cells at the time of co‐culture (Figure 1B). The number of iL in the presence of iTECs increased between day 0 and day 7 and waned thereafter (Figure 2A, 2B). Notably, large‐scale expansion (approximately 30‐fold) of iLs was dependent on the presence of iTECs, IL‐2 and IL‐7, although a small number of iLs emerged transiently in co‐cultures of HSC‐eBMCs and iTECs in the absence of cytokines (Figure 2A, 2B). Thus, iLs can be readily generated by 7‐day co‐culture of HSC‐eBMCs and iTECs in the presence of appropriate cytokines. Contrary, iL increased little in the absence of iTECs with or without the cytokines.

### 3.5 Analysis of the origin and phenotype of *in vitro*‐generated iLs

**FIGURE 3:**
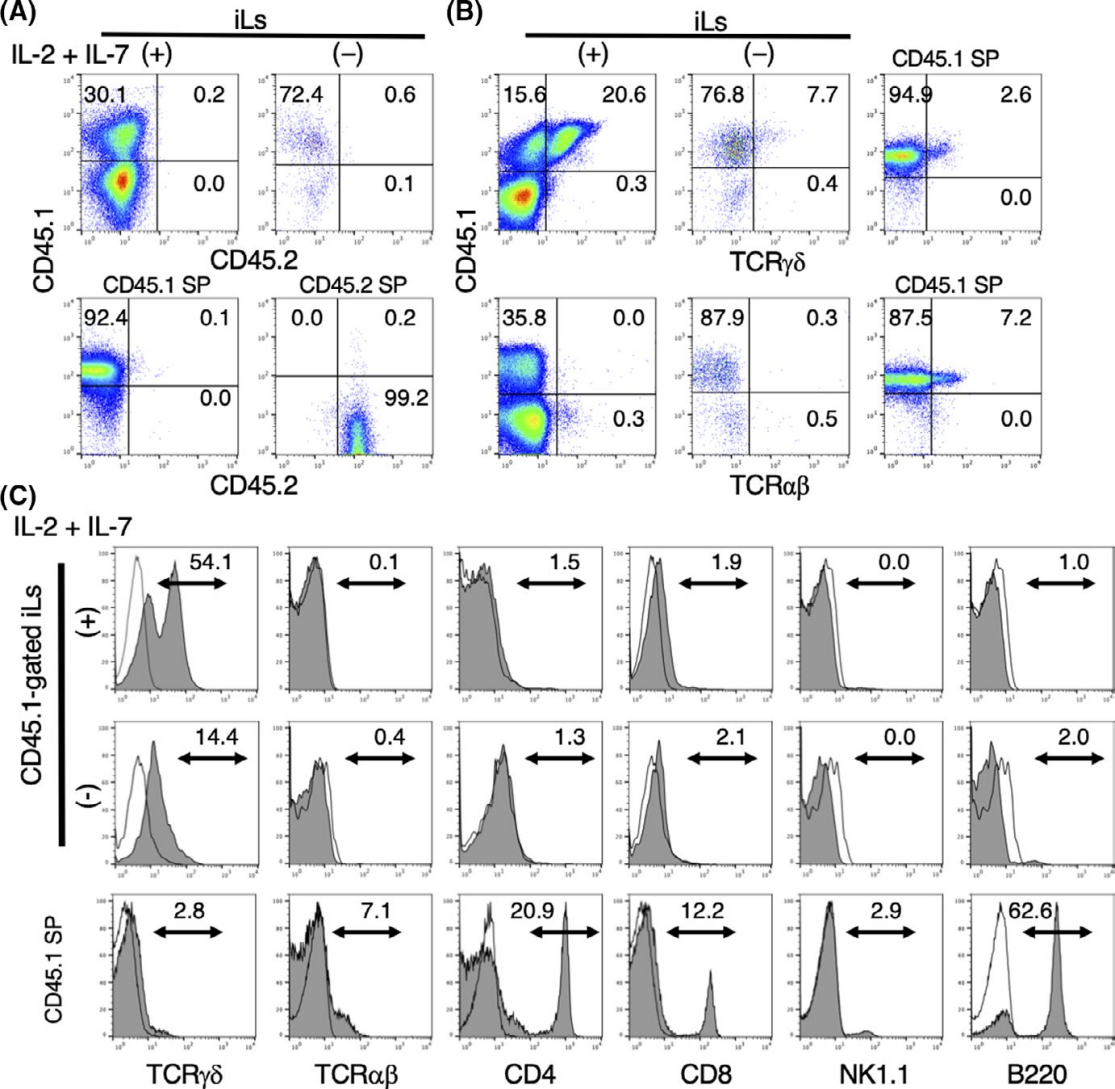
Flow cytometric analysis of the surface phenotype of induced lymphocytes (iLs) generated from HSC‐eBMCs and iTECs. (A) Top row: Expression of CD45.1 and CD45.2 on iLs derived from co‐culture of CD45.1^+^ HSC‐eBMCs and CD45.2^+^ iTECs in the presence (+) or absence (‐) of IL‐2 + IL‐7 for 7 days. Bottom row: Spleen cells from CD45.1 and CD45.2 congenic C57BL/6 mice were analysed as controls. (B) Left and middle columns: Expression of TCRγδ or TCRαβ on iLs cultured for 7 days as described for (A). Right column: Spleen cells from CD45.2 and CD45.1 congenic C57BL/6 mice were analysed as controls. (C) Top and middle rows: Expression of lymphocyte markers on CD45.1‐gated iLs cultured for 7 days as described for (A). Bottom row: Spleen cells from CD45.1^+^ C57BL/6 mice were analysed as controls. Open histograms represent the isotype controls, arrows indicate positive cells, and numbers indicate percentages

To assess the origin and subtypes of iLs derived from HSC‐eBMC +iTEC co‐cultures, we analysed the cells by flow cytometry for the expression of CD45.1, CD45.2, TCRαβ and TCRγδ, as well as T‐cell subset, B cell and NK cell markers. The iLs generated *in vitro* expressed CD45.1, but not CD45.2 (Figure 3A), which indicates that they originated from the HSC‐eBMCs, and not from the iPSCs. Interestingly, CD45.1^+^ TCRγδ^+^ cells represented approximately 21% and 8% of the iLs generated in the presence and absence, respectively, of IL‐2 + IL‐7 on day 7, whereas TCRαβ cells were virtually undetectable in the presence or absence of cytokines (0.0% and 0.3%, respectively; Figure 3B).

After gating of iLs on CD45.1^+^ cells, the preferential differentiation of TCRγδ^+^ cells from co‐cultures was even more striking. Thus, γδT cells and αβT cells represented 54.1% and 0.1% of CD45.1^+^ cells, respectively, in the presence of cytokines and 14.4% and 0.4%, respectively, in the absence of cytokines on day 7 (Figure 3C). Moreover, CD4^+^, CD8^+^, NK1.1^+^ and B220^+^ cells were virtually absent in the 7‐day co‐cultures (Figure 3C). Similar results were obtained when co‐cultures were examined on day 14 (data not shown). These data demonstrate that co‐culture of HSC‐eBMCs and iTECs with IL‐2 + IL‐7 preferentially results in the differentiation of TCRγδ cells, with relatively few mature αβT, NK or B cells.

### 3.6 Analysis of the *TCR*γδ repertoire of iLs

Next, we examined CD3 expression in CD45.1^+^ TCRγδ cells and additionally analysed the *Vγ* and *Vδ* TCR repertoire of the induced γδT cells. CD3 molecule is complexed with TCR and induces signals to intracytoplasmic domains following antigen recognition, which indicates that its expressing cells are functional T cells.^37^

**FIGURE 4:**
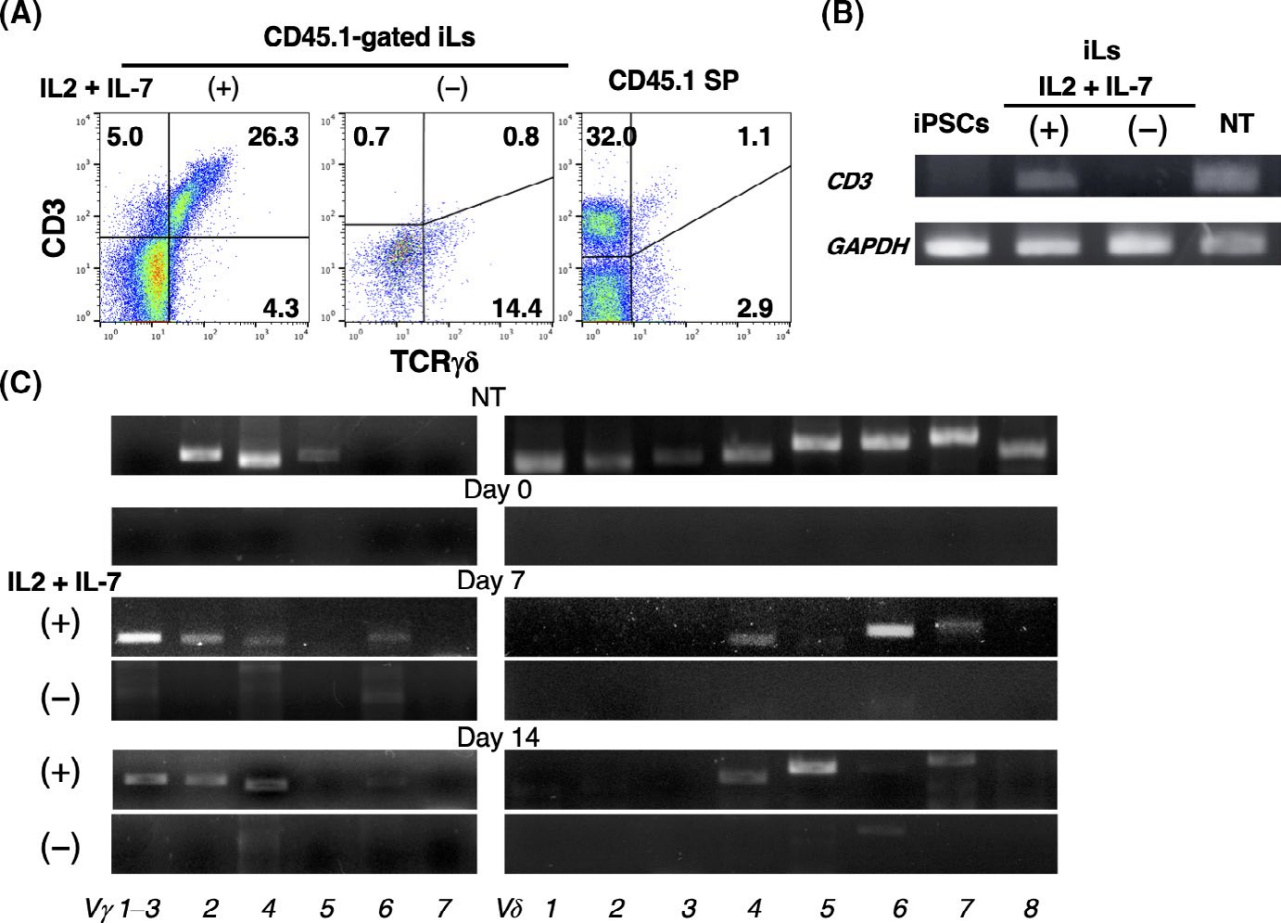
Expression of CD3 and the TCRγδ repertoire of induced lymphocytes (iLs) generated from HSC‐eBMCs and iTECs. (A) Expression of CD3 and TCRγδ on CD45.1‐gated iLs derived from co‐cultures of CD45.1^+^ HSC‐eBMCs and CD45.2^+^ iTECs in the presence (+) or absence (−) of IL‐2 + IL‐7 for 7 days. Spleen cells from CD45.1^+^ C57BL/6 mice were analysed as controls (right plot). Numbers indicate cell percentages. (B) RT‐PCR analysis of *CD3* and *GAPDH* (control) in iLs cultured as described in (A). (C) RT‐PCR analysis of the *TCR Vγ* and *Vδ* repertoire in HSC‐eBMCs (day 0), or iLs from co‐cultures incubated with or without IL‐2 + IL‐7 for 7 or 14 days. Mouse newborn thymus (NT) were analysed as a control in (B) and (C)

Flow cytometry of the CD45.1^+^ cells produced in IL‐2 + IL‐7–containing co‐cultures revealed that approximately 26% expressed both TCRγδ and CD3, whereas little CD3 expression was observed in the absence of the cytokines (Figure 4A). RT‐PCR analysis yielded the same results (Figure 4B). We next analysed the *TCR*γδ repertoire of the cells by RT‐PCR. iLs generated in the presence of IL‐2 and IL‐7 expressed *V*γ*1–3*, *2*, *4* and *6*, and *Vδ4*, *6* and *7* on day 7, and *Vγ1–3*, *2* and *4*, and *Vδ4*, *5* and *7* on day 14. Conversely, these components of *TCRγδ* were virtually absent in cells generated in the absence of the cytokines (Figure 4C). Similar results were observed in cells harvested after 21 days of co‐culture, although the expression levels were low compared with cells analysed on days 7 and 14 (data not shown). Thus, the induced *γδ*T cells expressed a broad *TCRγδ* repertoire reminiscent of that observed in T cells from neonatal mouse thymocytes.

### 3.7 Long‐term effects of transferred iLs in mice

**FIGURE 5:**
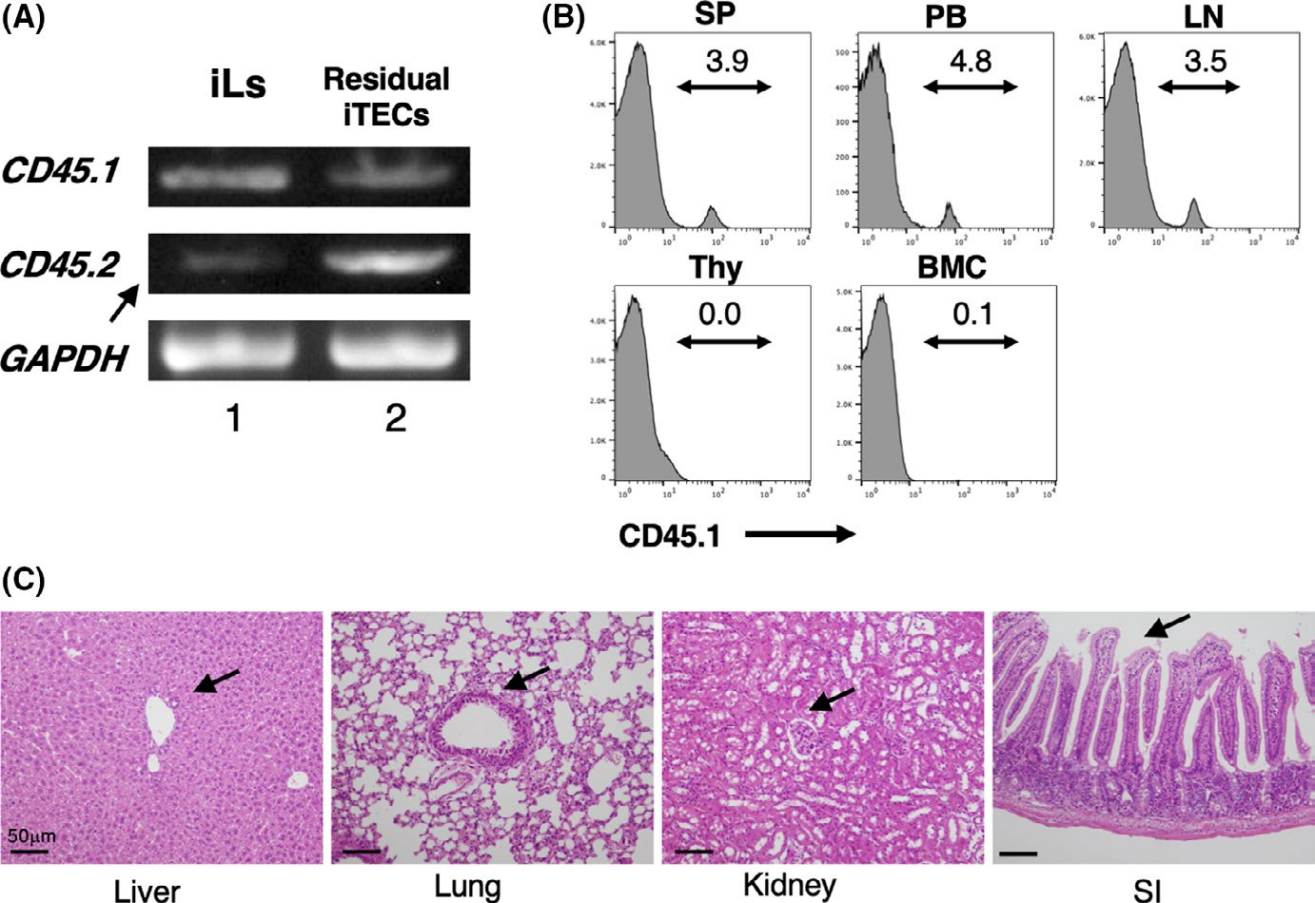
*CD45*.*1* and *CD45*.*2* allele typing of induced lymphocytes (iLs) and fate of iLs after transfer to mice. (A) PCR analysis of genomic DNA from iLs after co‐culture of CD45.1^+^ HSC‐eBMCs with CD45.2^+^ iTECs +IL‐2 + IL‐7 for 7 days, and from residual iTECs after harvest of the iLs. Arrow indicates a minor population of CD45.2^+^ iPSCs or iPSC‐derived iTECs. (B) Flow cytometric analysis of CD45.1^+^ cells isolated from the spleen (SP), peripheral blood (PB), small intestine lymph nodes (LN), thymus (Thy) and bone marrow (BMC) of CD45.2 C57BL/6 mice after autologous BMT and injection of CD45.1^+^ iLs for 14 days. (C) Histology of liver, lung. kidney and small intestine (SI) sections from mice on day 31 after BMT and iLs injection. Arrows indicate portal area in the liver, bronchus in the lung, glomerulus in the kidney and crypts in the SI. (H&E staining, ×200: bar indicates 50 µm.)

One potential drawback to the therapeutic use of iPSCs and activated lymphocytes is their ability to induce autoimmunity and/or tumorigenesis. Therefore, we next investigated the effects of iLs injected into CD45.2^+^ congenic mice that had undergone autologous BMT. RT‐PCR analysis of the iLs prior to injection confirmed that most cells were *CD45*.*1* ^+^ iLs with a small fraction of residual *CD45*.*2* ^+^ iPSCs or iPSC‐derived iTECs (Figure 5A). On day 14 after iL transfer, CD45.1^+^ cells were detectable in the recipient spleen, peripheral blood and small intestinal lymph nodes, but they were scarce or absent in the thymus and bone marrow (Figure 5B). Histological analysis of the organs on day 31 after iL transfer revealed essentially normal organs, with no specific findings in the liver, lung, kidney or small intestine (Figure 5C). In addition, these mice remained healthy and survived for >1 year (data not shown). These findings indicate that transfer of *in vitro*‐generated iLs, containing predominantly γδT cells with some residual iPSCs/iPSC‐derived cells, did not have any overt detrimental effects *in vivo*.

### 3.8 Effects of induced γδT cells on growth of leukemia

**FIGURE 6:**
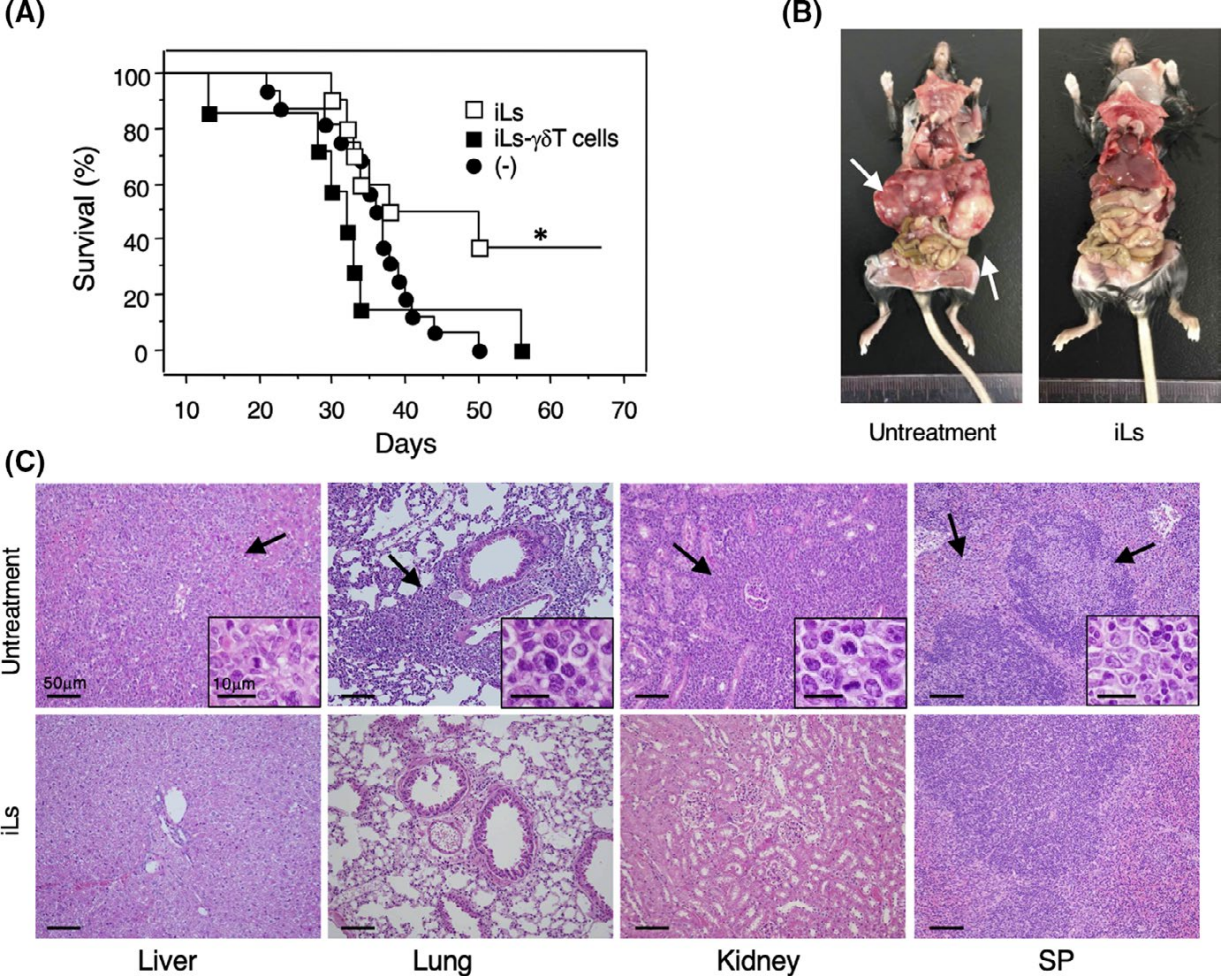
Effects of induced lymphocytes (iLs) and γδT‐depleted iLs in leukemia‐bearing mice. (A) Kaplan‐Meier survival curves of CD45.2 C57BL/6 mice injected with CD45.2^+^ EL‐4 leukemia cells followed by no treatment (−) (*n*=16), injection of 3 × 10^6^ iLs (*n* = 10) or injection of 3 × 10^6^ γδT‐depleted iLs (*n* = 7). *p < 0.05 versus untreated mice and mice injected with γδT‐depleted iLs by log‐rank test. (B) Representative macroscopic findings of leukemia‐bearing untreated or iL‐injected mice on day 50. Arrows indicate metastasis to the liver. (C) Representative histological findings of the liver, lung, kidney and spleen of leukemia‐bearing untreated (upper panel) or iL‐injected (lower panel) mice on day 50. Arrows point to abundant tumour cells observed in the organs of untreated mice (black arrows and insets) but not in the iL‐treated mice. (H&E staining, ×200: bar indicates 50 µm. Inset, higher magnification of the tumour cells: bar indicates 10 µm)

γδT cells have shown efficacy in the treatment of haematological malignancies and solid cancers in clinical trials.^38^, ^39^ Therefore, we investigated whether γδT cells in the iLs were functional and had anti‐tumour activity in mice. We injected C57BL/6 mice intravenously with EL‐4 leukemic cells (H‐2^b^, CD45.2) and then injected vehicle or iLs from 7‐day co‐cultures. To determine whether γδT cells contributed to the potential efficacy of iLs, we also included mice injected with iLs depleted of γδT cells (<1% residual γδT cells) (Fig. S3). All the mice transplanted with leukemic cells alone died within 50 days, whereas the survival rate of leukemic cell‐bearing mice injected with iLs was significantly prolonged, with 30% of these mice exhibiting long‐term survival (>100 days) (Figure 6A). Strikingly, depletion of γδT cells from the transferred iLs eradicated their ability to prolong mouse survival (Figure 6A), which demonstrates a primary role for γδT cells in the beneficial effects of the iLs. Macroscopic evaluation of the mice on day 50 revealed that metastases were mainly present in the liver of leukemia‐bearing mice (Figure 6B), and microscopic analysis revealed tumour cell infiltration into the lung, kidney and spleen in addition to the liver (Figure 6C). Conversely, mice injected with iLs had essentially normal livers, and few tumour cells were found when the organs were examined microscopically (Figure 6B, 6C). Analysis of mice that succumbed to leukemia despite injection of iLs revealed tumour cell infiltration into tissues that was comparable to the untreated leukemia‐bearing mice. Notably, however, tumour‐bearing mice injected with γδT cell‐depleted iLs showed a similar pattern of tumour growth to the untreated mice, namely tumour cell infiltration into all organs examined (data not shown). Collectively, these findings demonstrate that *in vitro*‐generated γδT cells have potent anti‐tumour effects *in vivo*.

## 4 DISCUSSION

In this study, we described a new method for the induction of γδT cells from HSC‐eBMCs and iPSC‐derived iTECs *in vitro*. We showed that iTECs and cytokines were critical for the differentiation of iLs from HSC‐eBMCs. Interestingly, the iLs were composed predominantly of γδT cells, with few αβT cells, B cells or NK cells, and the γδT cells expressed a broad repertoire of *Vδ* and *Vγ TCRs*. Finally, the *in vitro*‐generated γδT cells were active and showed a robust anti‐leukemia response *in vivo*, but had no detectable deleterious effects, including no evidence of autoimmunity or tumorigenesis. There have been few reports on the use of iPSCs as supporting cells to obtain the target cells, and our method thus adds a new application for iPSCs.

The results of the present study are consistent with a previous report on the induction of TECs from iPSCs *in vitro*.^20^ We observed comparable development of spindle/oval epithelial‐like cells, and the transcription factor expression patterns and growth rates were similar in both studies. These findings indicated that TECs differentiated from iPSCs and had matured for a period of 2 weeks, after which they achieved a steady state. We used the iTECs of the 14‐day period for co‐culture with HSC‐eBMCs, because the thymus shows the highest function at the newborn stage.^17^, ^18^

Using the CD45 congenic system, we found that γδT cells were induced from HSC‐eBMCs, and not iPSCs, and that IL‐2 and IL‐7 were required for the expansion of CD3^+^ γδT cells with a broad TCRγδ repertoire. Thus, whilst the presence of iTECs is critical for the development of γδT cells *in vitro*, the process is promoted by the addition of the appropriate cytokines.

In addition to T cells, other lymphocyte types develop or are present in the thymus, albeit in small numbers.^40^, ^41^ Despite this, we observed predominant induction of γδT cells in culture, with the production of few cells expressing TCRαβ, CD4, CD8, B220 or NK1.1. The reasons for the near‐exclusive induction of γδT cells are not yet clear. It is possible that additional activation and/or inhibition signals may be needed to generate other lymphocyte subsets,^42^, ^43^ especially because γδT cells develop before αβT cells *in vivo*.^7^, ^44^ Alternatively, it may be necessary to further modify the iTEC culture system to enable αβT cell development, such as changes to the three‐dimensional structure^13^, ^45^ and/or addition of distinct cortical/medullary iTEC types.

Generally, *Vγ4–6* ^+^ cells develop and migrate into the dermis and reproductive epithelium before birth, whereas *Vγ1*, *2*, *4* and *7* ^+^ cells are induced predominantly in lymphoid organs and the intestinal epithelium after birth in mice.^37^ Our co‐culture system showed that the induced γδT cells expressed *Vγ1–3*, *2*, *4* and *6*, and *Vδ4–7* ^+^ between days 7 and 14 in culture. This is likely to be due to differences between the generation of thymic environment *in vivo* and our system, such as the use of iTECs, which are postulated to be equivalent to newborn TECs, together with HSC‐eBMCs purified from adult mice. Nonetheless, the method described here is significant because it at least partly reproduces the environment necessary to obtain γδT cells from two types of stem cell‐derived cells *in vitro*.

Analysis of the fate of iLs after transfer to mice revealed their presence in the spleen, peripheral blood and intestinal lymph nodes, but not the thymus or bone marrow, of recipient mice. These findings indicate that the transferred cells are sufficiently mature to survive and migrate to peripheral hematopoietic organs. In addition, the recipient mice survived for >1 year and showed no apparent pathological effects of iLs, including the tumorigenesis and autoimmune disease. This important observation suggests that the transfer of γδT cells is not harmful, even if they contain low numbers of iPSCs or differentiated iTECs.

Another important conclusion from this study is that γδT cells are effectively able to suppress leukaemia growth *in vivo*, as has been shown in clinical trials for some hematopoietic malignancies.^39^ Leukemic mice transplanted with iLs showed significantly reduced tumour growth and prolonged survival compared with untreated mice or those transplanted with γδT cell‐depleted iLs. These findings suggest that γδT cells played the dominant role in inhibiting leukemic cell growth *in vivo*. Further studies will be necessary to determine the detailed mechanism of action of the γδT cells.

iPSCs have been used to generate differentiated cells for use in regenerative medicine in humans, and patient‐derived iPSCs have also proven extremely useful for *in vitro* function/toxicity screening during drug development.^46^, ^47^ In this study, we used iPSC‐derived cells to support the differentiation of specific target cells, thereby identifying a third possible use for iPSCs. Of note, although we observed no autoimmunity or malignancy in animals injected with iL populations containing low levels of residual iPSCs/iPSC‐derived cells, it will be important to monitor this as part of the safety/efficacy evaluation of iPSC‐derived cells as potential therapeutics for use in humans.

In summary, we present a method for the induction of γδT cells using iPSC‐derived iTECs co‐cultured with HSC‐eBMCs. The method may be useful for developing treatments for several intractable diseases in humans, including malignant tumours. Moreover, given that the functions of γδT cells are not MHC‐restricted,^9^ stocks of either MHC‐matched or unmatched iPSCs and/or BMCs could be deposited in cell banks for use in transplantation, thus reducing the need for patient‐derived cells.^48^, ^49^

## CONFLICT OF INTEREST

All authors declare that no conflicts of interest exist.

## AUTHOR CONTRIBUTIONS

**Naoki Hosaka:** Conceptualization (lead); Data curation (lead); Formal analysis (lead); Funding acquisition (lead); Investigation (lead); Methodology (lead); Project administration (lead); Writing‐original draft (lead). **Seiji Kanda:** Methodology (equal); Project administration (supporting); Supervision (equal). **Takaki Shimono:** Methodology (supporting); Project administration (supporting); Visualization (supporting). **Toshimasa Nishiyama:** Project administration (supporting); Supervision (lead).

## Supporting information

Supplementary Material

## Data Availability

Supporting data are available from the corresponding author upon reasonable request.
