# Supplementary material for: Induction of γδT cells from HSC‐enriched BMCs co‐cultured with iPSC‐derived thymic epithelial cells
Source: J Cell Mol Med. 2021 Oct 23;25(22):10604–13. doi: 10.1111/jcmm.16993 (PMC8581322; doi:10.1111/jcmm.16993)
Supplement: Supplementary file 1 — Supplementary Material [file JCMM-25-10604-s001.pdf]

Supplemental information

Induction of γδT cells from HSC-enriched BMCs co-cultured with iPSC-derived thymic epithelial cells

Naoki Hosaka, Seiji Kanda, Takaki Shimono, and Toshimasa Nishiyama

SUPPLEMENTAL MATERIAL

Supplemental figures

Figure S1. Related to Figure 2. RT-PCR analysis of *CD45* alleles expressed in iPSCs.

Figure S2. Related to Figure 2. Flow cytometric analysis of CD34, CD117, CD45.1, TCR$\gamma\delta$, and TCR$\alpha\beta$ expression in BMCs and HSC-eBMCs.

Figure S3, Related to Figure 6. Flow cytometric analysis of iLs after depletion of TCR$\gamma\delta^+$ cells.

Supplemental Tables

Table S1. Primers used for RT-PCR
Table S2. Primers used for PCR

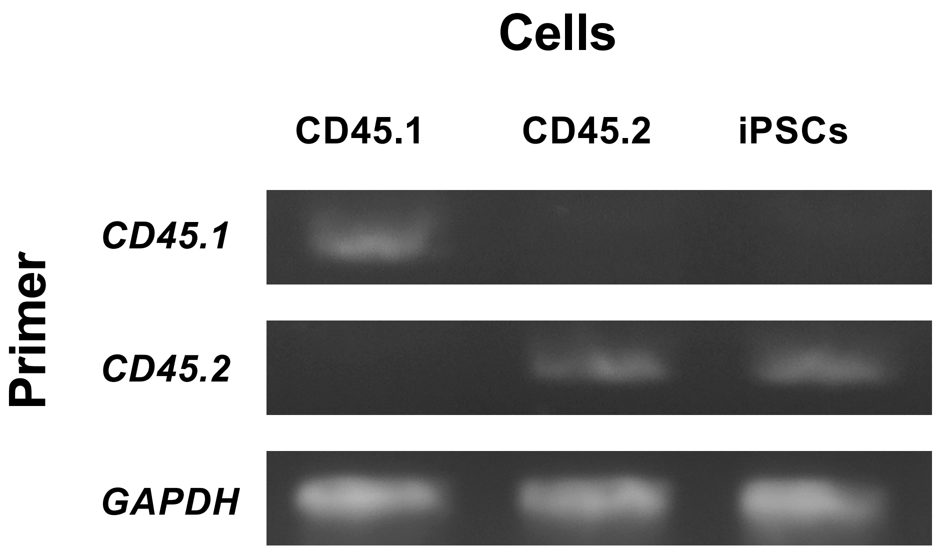

Figure S1. Related to Figure 2. RT-PCR analysis of *CD45* alleles expressed in iPSCs. Genomic DNA isolated from iPSCs and spleen cells from CD45.1 and CD45.2 C57BL/6 congenic mice were analyzed using *CD45.1*- and *CD45.2*-specific primers. *GAPDH* was analyzed as a control.

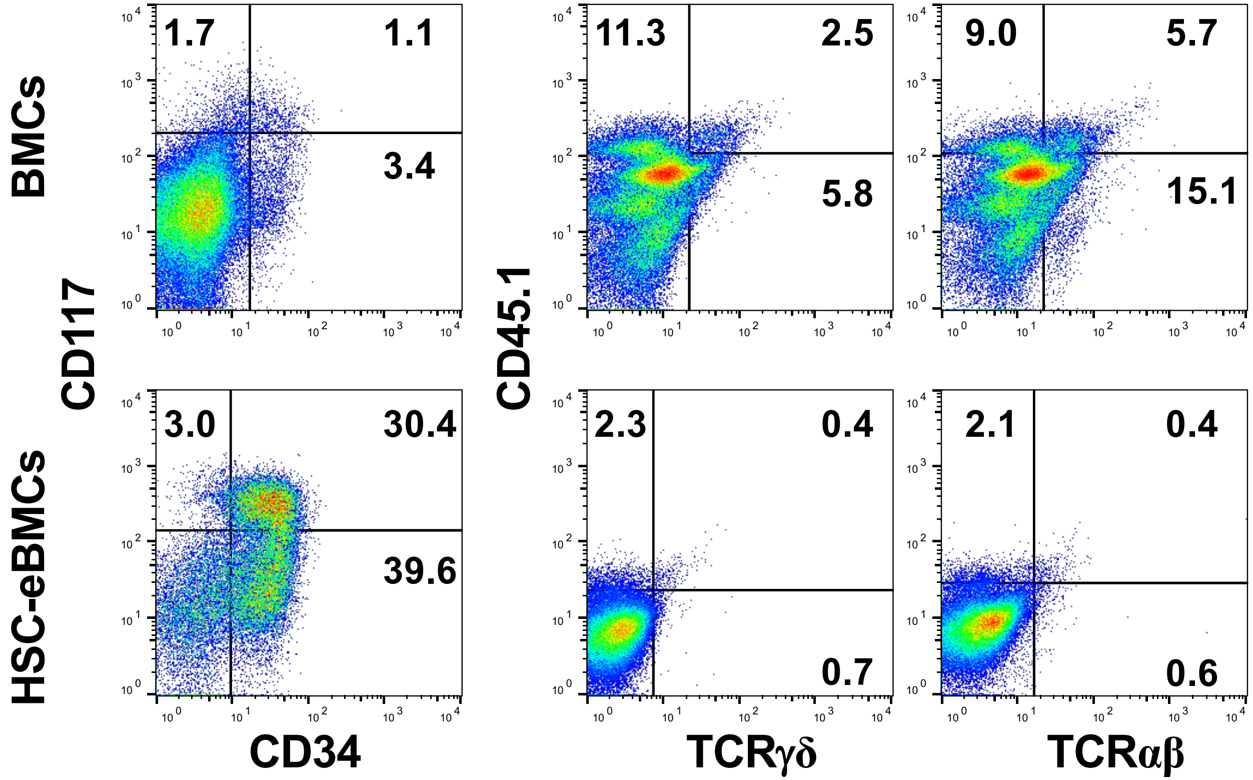

Figure S2. Related to Figure 2. Flow cytometric analysis of CD34, CD117, CD45.1, TCRγδ, and TCRαβ expression in BMCs and HSC-eBMCs.

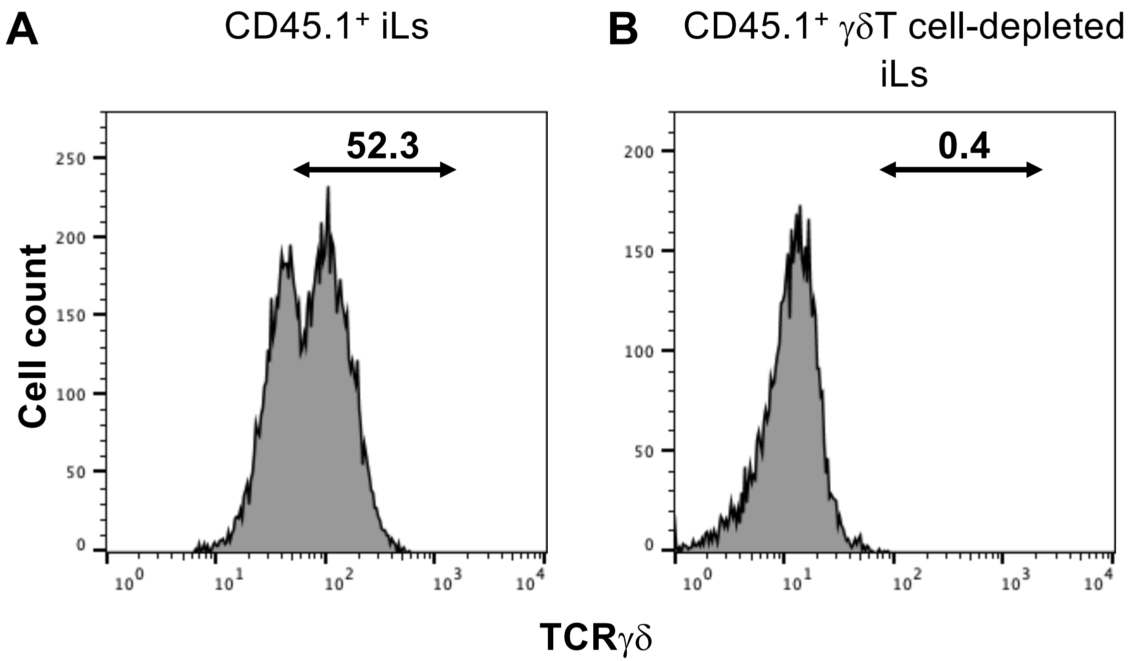

Figure S3, Related to Figure 6. Flow cytometric analysis of iLs after depletion of TCRγδ⁺ cells. iLs were generated *in vitro* by co-culture of iTECs and HSC-eBMCs. iLs were depleted of γδT cells by incubation with streptavidin-conjugated anti-TCRγδ mAb GL3 and biotin-conjugated immunobeads, and expression of TCRγδ in the input iL (A) and γδT cell-depleted iL populations (B) was analyzed by staining with FITC-conjugated anti-TCRγδ mAb UC7. Numbers indicate % TCRγδ⁺ cells.

Supplemental Tables

Table S1. Primers used for RT-PCR

Gene	Sequences
GAPDH	(F) 5′-ACC ACA GTC CAT GCC ATC AC-3′
	(R) 5′-TCC ACC ACC CTG TTG CTG TA-3′
Nanog	(F) 5′-AAG TAC CTC GAC CTC CAG CA-3′
	(R) 5′-CGT AAG GCT GCA GAA AGT CC-3′
Edh1	(F) 5'-TCA GGG AAC ATT CAT GCC AC-3’
	(R) 5'-TTC TAT GCC GTC TCC ATC AAC-3'
Hoxa3	(F) 5′-CAC CTG GAA CTG GAG ACC AT-3′
	(R) 5′-ACC GTA GAT CGC TGA GCT GT-3′
Fgfr2IIIb	(F) 5'-CAC CGA GAA GAT GGA GAA GC-3’
	(R) 5'-GTC TGA CGG GAC CACA CTT T-3’
Krt5	(F) 5′-TGG GAC AGG AAG AGA GGT GAT C-3′
	(R) 5′-ACC AAA ACC AAA TCC ACT GCC G-3′
Krt8	(F) 5′-CTC ATC AAG AAG GAT GTG GAC-3′
	(R) 5′-TAC ATG GTT TCA GCC TCA GCT-3′
Pax1	(F) 5′-GCA GCC GGC TAC CTA TCT C-3′
	(R) 5′-GGC AGT CCG TGT AAG CTA CC-3′
Pax9	(F) 5′-GCT GCC CTA CAA CCA CAT TT-3′
	(R) 5′-ACC AGA AGG AGA GCA GCA CTG T-3′
Plet1	(F) 5'-CTT CCA CAC CTG GGA CTG TT-3’
	(R) 5'-CGT CCT CCT TCA CTG CTT TC-3’

Aire	(F) 5′-CAA CTC TGG CCT CAA AGA GC-3′
	(R) 5′-CCT GAC TCA AAC ACC TGC TG-3′
FoxN1	(F) 5′-ACT CTT CCC AAA GCC CAT CT-3′
	(R) 5′-AGC AAT GGG GTC TTT CCT CT-3′
CD3ε	(F) 5′-GAG CTG GCT GCG TCC GCC AT-3′
	(R) 5′-CGC TGG CCT TTG CGG ATG GG-3′
Common γ	5′-CTT ATG GAG ATT TGT TTC AGC-3′
Vγ1-3	5′-ACA CAG CTA TAC ATTGGT AC-3′
Vγ2	5′-CGG CAA AAA ACA AAT CAA CAG-3′
Vγ4	5′-TGT CCT TGC AAC CCC TAC CC-3′
Vγ5	5′-TGT GCA CTGGTA CCA ACT GA-3′
Vγ6	5′-CTC CAA AGA ATG CTG TGT AG-3′
Vγ7	5′-AAG CTA GAG GGG TCC TCT GC-3′
Common δ	5′-CGA ATT CCA CAA TCT TCT TG-3′
Vδ1	5′-ATT CAG AAG GCAACA ATG AAA G-3′
Vδ2	5′-AGT TCC CTG CAG ATC CAAGC-3′
Vδ3	5′-TTC CTG GCT ATT GCC TCT GAC-3′
Vδ4	5′-CCGCTT CTC TGT GAA CTT CC-3′
Vδ5	5′-CAG ATC CTT CCAGTT CAT CC-3′
Vδ6	5′-CTT AGT GGA GAG ATG GTT TT-3′
Vδ7	5′-CGC AGA GCT GCA GTG TAA CT-3′
Vδ8	5′-AAG GAA GAT GGA CGA TTC AC-3′

Table S2. Primers used for PCR

Gene	Sequences
Common Ly5	5′-CAG GTC CAC TGA AAC AGA GGA TG-3′
Ly5.1	5′-CTG AGC CTG CAT CTA AAC CTG ATC-3′
Ly5.2	5′-CTG AGC CTG TAT CTA AAC CTG AGT-3′
